# Targeting the EphA2 pathway: could it be the way for bone sarcomas?

**DOI:** 10.1186/s12964-024-01811-7

**Published:** 2024-09-09

**Authors:** Giorgia Giordano, Cristina Tucciarello, Alessandra Merlini, Santina Cutrupi, Ymera Pignochino

**Affiliations:** 1 Sarcoma Unit, Candiolo Cancer Institute, FPO-IRCCS, 10060 Candiolo, TO Italy; 2 Department of Oncology, University of Turin, 10043 Orbassano, TO Italy; 3 Department of Clinical and Biological Sciences, University of Turin, 10043 Orbassano, TO Italy

**Keywords:** EphA2, Bone sarcomas, Targeted therapy, Immunotherapy, Drug delivery

## Abstract

Bone sarcomas are malignant tumors of mesenchymal origin. Complete surgical resection is the cornerstone of multidisciplinary treatment. However, advanced, unresectable forms remain incurable. A crucial step towards addressing this challenge involves comprehending the molecular mechanisms underpinning tumor progression and metastasis, laying the groundwork for innovative precision medicine-based interventions. We previously showed that tyrosine kinase receptor Ephrin Type-A Receptor 2 (EphA2) is overexpressed in bone sarcomas. EphA2 is a key oncofetal protein implicated in metastasis, self-renewal, and chemoresistance. Molecular, genetic, biochemical, and pharmacological approaches have been developed to target EphA2 and its signaling pathway aiming to interfere with its tumor-promoting effects or as a carrier for drug delivery. This review synthesizes the main functions of EphA2 and their relevance in bone sarcomas, providing strategies devised to leverage this receptor for diagnostic and therapeutic purposes, with a focus on its applicability in the three most common bone sarcoma histotypes: osteosarcoma, chondrosarcoma, and Ewing sarcoma.

## Introduction

Bone sarcomas belong to a rare and heterogeneous group of malignant mesenchymal primary tumors originating from the osseous tissue, representing less than 1% of all malignancies [1]. Osteosarcoma (OS) and Ewing sarcoma (ES) occur mainly in adolescents and young adults, while chondrosarcoma (CS) has a peak incidence in the seventh decade of age [2]. Surgical excision of the tumor combined with radiotherapy and chemotherapy can achieve good results as first-line treatment; however, 40% of patients affected by bone sarcomas still die of the disease due to poor histological response to therapy, onset of multiple metastases, and relapses [3]. Several targeted therapies and immunotherapies against advanced unresectable bone sarcomas have been investigated in preclinical and clinical studies. Still, among innovative agents, only mifamurtide has entered the clinical management of OS until now [4, 5]. Identifying novel molecular targets will be instrumental in overcoming the current impasse in treating relapsing disease. Receptor tyrosine kinases (RTKs) have been extensively studied as therapeutic targets in bone sarcoma, being implicated in several steps of their onset and progression [6–13]. Among them, the erythropoietin-producing hepatocellular (Eph) tyrosine kinases receptor family member 2 (EphA2) is a driver oncofetal protein implicated in self-renewal, chemoresistance, and metastasis [14–16] so that its targeting is now under preclinical and clinical investigation [17–21]. We previously showed that bone sarcoma patient-derived models overexpressed EphA2 and are sensitive to its inhibition [7, 13]. In the present work, we combined a thorough overview of the literature to pursue the current knowledge of EphA2 functions in different tumors, focusing on bone sarcomas, and the main strategies developed so far for its specific targeting, exploring its potential applicability in these settings.

## Eph receptors signaling and ephrins

Eph receptors belong to a large subfamily of RTKs expressed in various cell types in developing and mature tissues [22, 23]. They have a highly conserved overall structure and are subdivided into two classes: EphA and EphB, based on their extracellular domain sequence homology and ligand binding specificity. In humans, nine EphA (A1-8, A10) and five EphB (B1-4, B6) receptors were identified with eight related ligands (Ephrin-A1-5 and Ephrin-B1-3) [24, 25]. The EphA receptor members bind preferentially to Ephrin-A ligands, while the EphB receptors bind to Ephrin-Bs, with some exceptions of cross-class binding such as EphA4, which interacts with Ephrin-B2 and B3, while EphB2 with Ephrin-A5 [26].

**Fig. 1:**
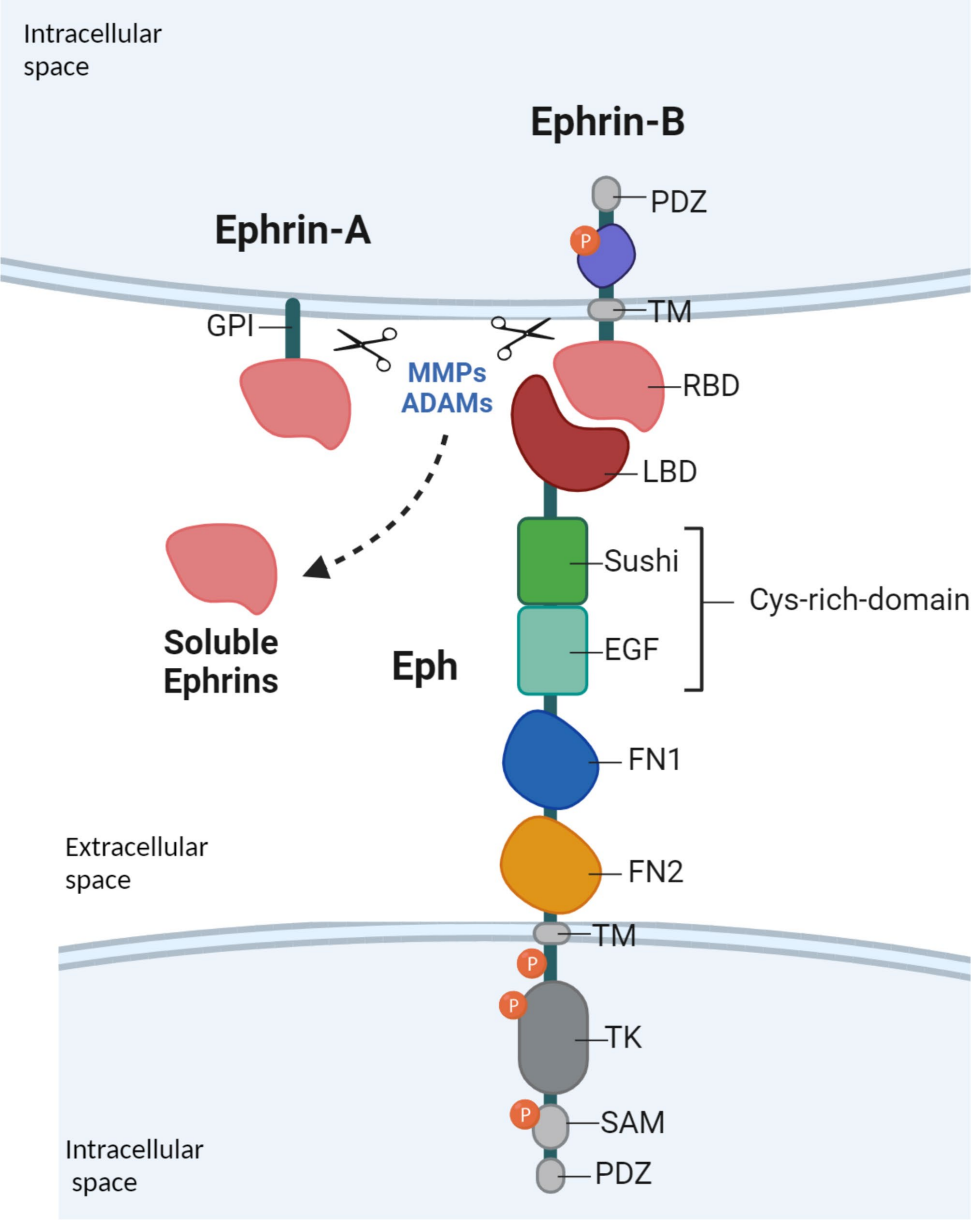
Schematic representation of the structural and functional domains of Eph receptor and its ligands. Eph receptors are single transmembrane proteins constituted by an extracellular and an intracellular side. The extracellular side is composed of a ligand-binding domain (LBD), a Cys-rich domain made of the Sushi and the Epidermal Growth Factor (EGF)-like domains, and two fibronectin (FN1 and FN2) domains. The intracellular side is composed of the transmembrane (TM) region, the tyrosine kinase (TK) domain, the Sterile Alpha Motif (SAM), and the PDZ domain. Ephrin ligands are constituted by a receptor-binding domain (RBD). Class A Ephrins are linked to the membrane through the GPI linkage, while class B Ephrins have a TM domain and an intracellular tail ending with a PDZ domain. Ephrins can be released from the cell surface by proteolytic cleavage done by proteases such as MMPs and ADAMs and can activate Eph receptors in a paracrine manner. *(Created with BioRender.com)*

Eph receptors are single transmembrane proteins constituted by an extracellular side, which exerts ligand-binding activity and an intracellular side with intrinsic enzymatic properties [26]. Starting from the N-terminal, the extracellular side of Eph receptors is composed of a ligand-binding domain (LBD) followed by a Cys-rich domain composed of the Sushi and the Epidermal Growth Factor (EGF)-like domains, and two fibronectin (FN) domains. The intracellular part of the Eph receptor is composed of the transmembrane (TM) region, the tyrosine kinase (TK) domain, the Sterile Alpha Motif (SAM), and the common structural PDZ domain (Fig. 1). The Eph ligands, Ephrins, are anchored to the cell membrane of interacting cells and share a conserved extracellular N-terminal receptor-binding domain (RBD) [25]. Class A is linked to the membrane through a glycosylphosphatidylinositol (GPI) linkage, whereas class B has a TM domain and an intracellular tail ending with a PDZ domain [24]. The interaction between Eph receptors and their ligands leads to the phosphorylation of various tyrosine (Tyr) residues between the TM and SAM domains. These post-translational modifications are crucial for the occurrence of the biological responses triggered by Eph signaling (Fig. 1).

**Fig. 2:**
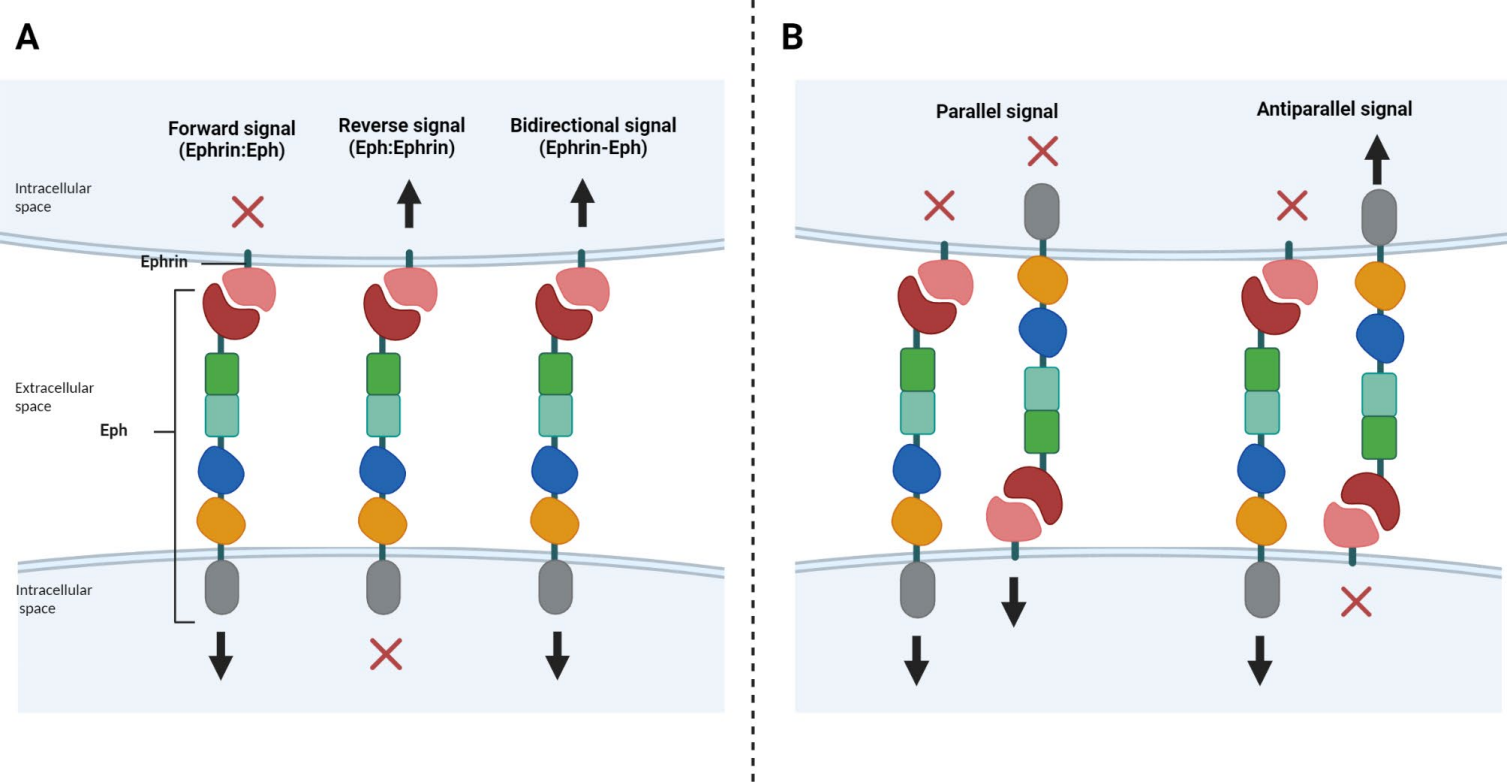
Schematic representation of the directional signaling evoked by Eph-Ephrin binding between adjacent interacting cells. (**A**) Eph receptor interacts with its ligand Ephrin on the adjacent cell, initiating a bidirectional signal, “forward” or “reverse”, based on the direction of signal flow. (**B**) The signal could also be “parallel” or “antiparallel”, if the Eph-Ephrin complex transmits the signal in the same or opposite directions, respectively. *(Created with BioRender.com)*

Physiologically, the Eph-Ephrin signaling pathway intervenes in multiple biological events such as axon guidance, tissue patterning, and blood vessel development in embryonic cells [27, 28]. It is strictly dependent on cell type and microenvironment and works bi-directionally. Namely, it can affect both the receptor-expressing and the ligand-expressing cells [4, 22, 24]. Indeed, when the Eph receptor interacts with its ligand Ephrin on the adjacent cell, it initiates bidirectional signaling, which can be categorized as “forward” or “reverse” depending on the direction of signal flow. The classical forward signal (Ephrin: Eph) is often cell repulsive, dependent on Eph kinase activity and it propagates in the Eph receptor-expressing cell; conversely, the reverse signal (Eph: Ephrin) is dependent on Fyn, a kinase belonging to the Src family, and it propagates in the Ephrin-expressing cell [27, 28] (Fig. 2A). Furthermore, as both Eph receptors and Ephrins can function concurrently as receptors and ligands when present on opposing cells, we can distinguish between simultaneous parallel or antiparallel signaling based on the direction of signal propagation. Signaling is deemed “parallel” if the Eph-Ephrin complex transmits the signal in the same direction and “antiparallel” if it transmits the signal in opposite directions [22, 29] (Fig. 2B).

Proteolytic cleavage serves as a feedback mechanism of this signaling process. However, the extracellular portions of shed Eph and Ephrin can interact with distant cells autonomously, independent of cell-cell contact, resulting in paracrine effects [30, 31]. For instance, they may act as monomeric inhibitors of bidirectional signaling [32]. Alternatively, cleaved ligands activate Eph receptors in an endocrine way. Soluble A-type Ephrin oligomers produced by the cleavage of GPI-anchored Ephrin-A1 on the plasma membrane by matrix metalloproteases (MMPs) or proteases of the A disintegrin and metalloproteinase (ADAM) family have the potential to activate EphA receptors, disrupting cell-cell contacts and increasing endothelial permeability which facilitates tumor metastasis to lungs [33, 34]. Soluble B-type Ephrins are involved in pathological conditions such as fibrosis and cancers [35] (Fig. 1).

## EphA2 is an oncofetal protein

Among Eph receptors, EphA2 is the most widely overexpressed in different tumor types [29]. EphA2 is a 130 kDa transmembrane glycoprotein of 976 amino acids encoded by the gene *EPHA2* in the human genome on chromosome 1p36 [36]. EphA2 could be described as a fetal oncoprotein since it physiologically plays key roles in several biological processes during development, including embryonic lens and inner ear formation, mammary epithelial branching morphogenesis, kidney development, and bone homeostasis, and it is aberrantly reactivated in several solid tumors [37–40].

EphA2 interacts with all the Ephrin-A family ligands with preferential binding to Ephrin-A1 (EFNA1), a Tumor Necrosis Factor (TNF)-α–inducible gene product [41]. During embryogenesis, its physiological functions following cell-cell contact rely on the binding with EFNA1 on the neighboring cells generating both reverse and forward signaling [27, 42]. During oncogenesis, EphA2 is overexpressed and signal transduction leads to modifications in cytoskeleton dynamics, cell adhesion, migration, metastasis, proliferation, and angiogenesis [43].

**Fig. 3:**
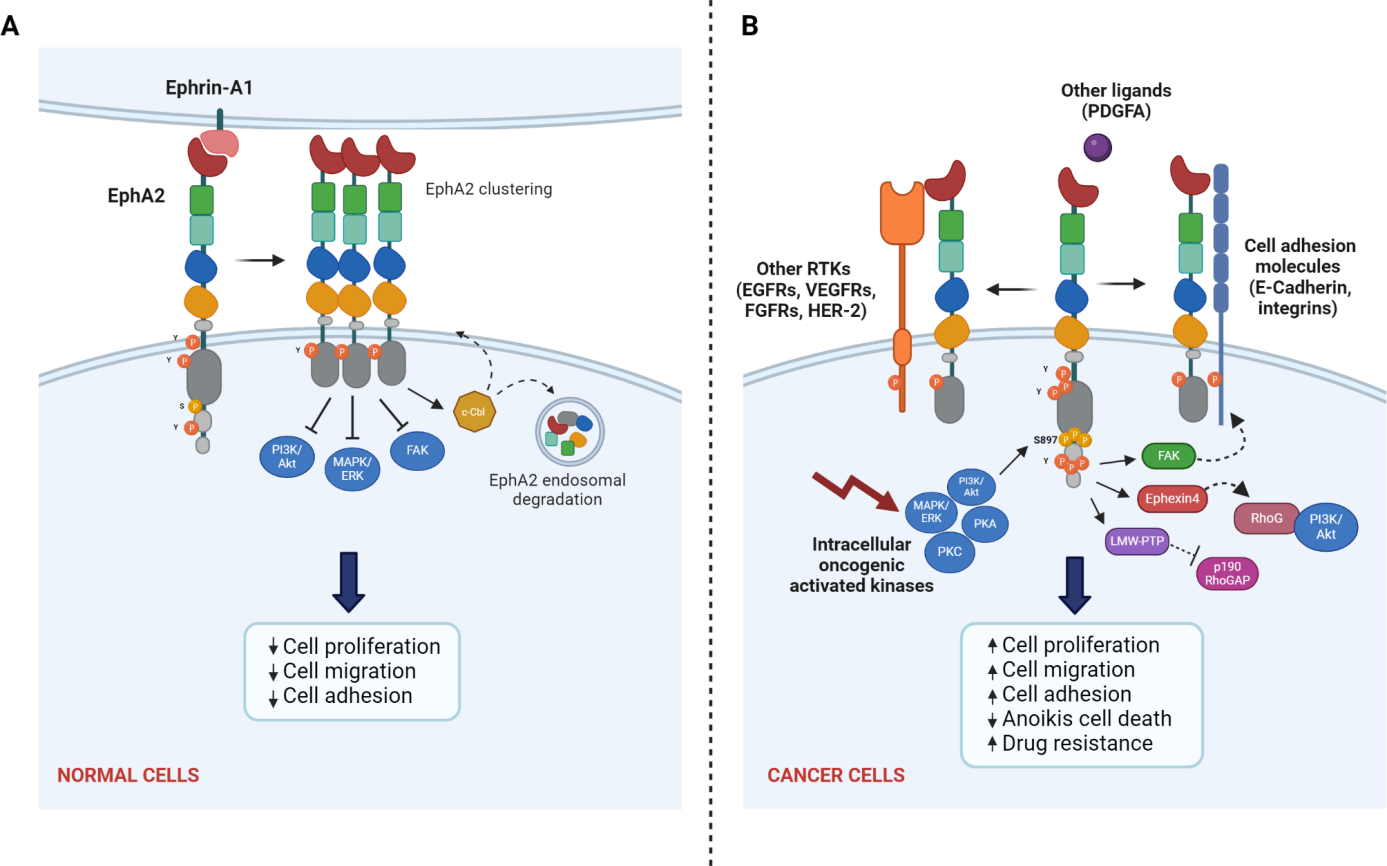
Ligand-dependent and ligand-independent EphA2 signal transduction, in normal and cancer cells, respectively. (**A**) In normal cells, the ligand-dependent EphA2 activation induced Tyrosine (Y) and Serine (S) phosphorylation, the Epha2 clustering, suppresses the proliferation, survival, and migration signaling pathways (ERK, Akt, FAK) inhibiting cell proliferation, resistance to apoptosis, and migration, and activates c-Cbl -mediated endosomal degradation and EphA2 recycling (**B**) In cancers, the non-canonical ligand-independent activation of EphA2 occurs through the dimerization with other RTKs (EGFR, HER2, FGFRs, VEGFRs) or cell adhesion molecules (E-cadherin, integrins); the direct binding with other ligands such as growth factors (e.g. platelet-derived growth factor A, PDGFA); or is mediated by intracellular oncogenic kinases (Akt, PKA, PKC, ERK). This induces the phosphorylation of Ser897, promoting cell proliferation, adhesion, and migration, other than drug resistance, protection to apoptosis and anoikis. *(Created with BioRender.com)*

During normal development, the ligand-dependent EphA2 activation suppresses the Extracellular signal-regulated Kinases (ERKs), the Akt, and Focal Adhesion Kinase (FAK) signaling pathways inhibiting cell proliferation, resistance to apoptosis, and migration [44] (Fig. 3A). However, in cancers, EphA2 is involved in a non-canonical activation through different mechanisms: (i) the dimerization with other RTKs such as the Epidermal Growth Factor Receptor (EGFR); the Human Epidermal Growth Factor Receptor-2 (HER2) [45, 46]; some members of the Fibroblast Growth Factor Receptors (FGFRs); and Vascular Endothelial Growth Factor Receptors, (VEGFRs), or with cell adhesion molecules (e.g. E-cadherin and integrins) [47, 48]; (ii) the direct binding with growth factors (e.g. the Platelet-Derived Growth Factor subunit A, PDGFA [49]; iii.) the direct phosphorylation of Serine-897 (P-Ser897 EphA2) located between the TK and SAM intracellular domains by intracellular oncogenic activated kinases, such as ERK Akt, and the Ribosomal S6 Kinase (RSK) [50–52]. P-Ser897 EphA2 recruits Ephexin4, a guanine nucleotide exchange factor, promoting resistance to the extracellular matrix detachment induced-cell death (anoikis), engaging the small GTPase Ras Homolog Gene Family Member G (Rho G)-Akt pathway activation (Fig. 3B) [53, 54]. EphA2 also plays a key role in integrin-mediated cell adhesion and migration through its association with FAK. In prostate cancer cells, the constitutive active EphA2/FAK complex is disassembled by the treatment with soluble EFNA1 that stimulates EphA2 Tyrosine phosphorylation and FAK dephosphorylation leading to the complex disassemble and the inhibition of cell migration [55, 56]. However, the inhibitory effect of EFNA1-induced tyrosine phosphorylation of EphA2 is reversed by the action of the Low Molecular Weight Phospho-Tyrosine Phosphatase (LMW-PTP), a protein frequently overexpressed in cancer [57]. Moreover, LMW-PTP inhibits the p190 RhoGAP, (a Rho-GTP inhibitor), destabilizing adherent junctions via a RhoA-dependent mechanism inducing cell detachment and migration [58] (Fig. 3B).

The involvement of EphA2 in several solid tumors including melanoma, bone sarcomas, glioblastoma, lung, colorectal, prostate, pancreatic, endometrial, breast, and gastric cancers is widely demonstrated [16, 59–72]. In these tumors, EphA2 was shown to correlate with tumor stages, cancer aggressiveness, metastatic potential, and poor patient survival, so that could be considered a biomarker of poor prognosis [73–77]. Moreover, some evidence has recently shown that EphA2 is implicated in tumor-microenvironment crosstalk, promotion of metastasis, and chemoresistance [17, 78–81]. EphA2 has been correlated with cancer-associated fibroblasts (CAFs) in many tumors. In gastric cancer, CAFs promote tumorigenesis through the EphA2 signaling pathway, and the treatment with the selective EphA2 inhibitor (ALW-II-41-27) or EphA2 silencing decreased the CAF-induced tumorigenesis [82]. Moreover, the EphA2-PI3K signaling pathway is involved in the CAF-induced vascular mimicry inducing gastric cancer cells to generate an endothelial-free blood delivery channel [83]. In addition, EphA2 may promote tumor-induced endothelial cell migration and angiogenesis, through its direct interaction with Caveolin-1 (CAV-1) and the consequent Akt activation and basic FGF (bFGF) production [84].

## EphA2 as a potential therapeutic target in bone sarcoma

In the realm of rare tumors, bone sarcomas remain a particularly challenging category, often labeled as “drug-orphan tumors”, because of the lack of effective treatments for advanced disease. This is mainly due to their heterogeneity and rarity, which pose significant obstacles to drug discovery and the development of innovative therapy. In this context, exploring EphaA2 targeting emerges as a promising avenue [85].

Our group has provided evidence suggesting that EphA2 might be an effective molecular target in the three main bone sarcomas (OS, ES, and CS). Through a combination of in silico analyses, and in vitro experiments using bone sarcoma cell lines and patient-derived xenografts, we observed significant overexpression of EphA2 compared to healthy controls [13]. Furthermore, our studies revealed that inhibiting EphA2 directly with ALW-II-41-27 or decreasing its expression impaired bone sarcoma growth [7, 13].

### EphA2 in osteosarcoma

OS is the most common primary bone tumor [3]. It mainly affects children and young adults under 25 years old. Still, a second peak of incidence usually arises at the age of 60 years with a frequency of 0.2–0.3 and 0.8/1.1 cases per 100,000 people per year in the general population and at the age of 15–19, respectively, and with a 1.4 male/female ratio [86–88]. There are 3 main types of primary osteosarcoma: intramedullary, juxtacortical, and extraskeletal osteosarcoma. The intramedullary osteosarcoma develops in the medullary cavity of a long bone is the most common (80%) and can be further divided into osteoblastic, condroblastic, fibroblastic, small-cell, and epithelioid based on main cell types. The juxtacortical osteosarcoma (10–15%) develops on the outer surface of the bones or the periosteum. The extraskeletal osteosarcoma is the rarest (< 5%) originates in soft tissues and can be induced by radiotherapy. Differences in clinical behavior and treatment choice for each subtype were exhaustively reviewed elsewhere [89]. OS is characterized by complex genetic changes and instability which result in recurrent amplifications and DNA copy number variations at different chromosomal regions [90]. Two recurrent somatic mutations implicated in the genesis of this tumor result from the progressive accumulation of genetic defects: a mutation in the Retinoblastoma Protein 1 (RB1) gene associated with retinoblastoma at 13q14 and mutations in the Tumor Protein 53 (TP53) gene associated with Li-Fraumeni syndrome at 17p13 [91]. These known tumor suppressor genes are key players in bone oncogenesis [92]. However, efficient targetingof RB1 and TP53 alterations remains elusive, and druggable gene alterations are still lacking. The standard therapeutic approach in localized OS combines surgery and polychemotherapy; in advanced disease with lung metastases, repeated lung metastasectomy can achieve a permanent cure in a subset of selected patients [93] but the overall prognosis remains dismal [94]. Fritsche-Guenther et al. showed a high overexpression of the EphA2 and its ligand in human OS samples analyzed by microarray [62]. Though EFNA1 was strongly upregulated in tumor tissues, it was also detected in fetal and normal adult human bone tissue. On the contrary, an EphA2 de novo expression has been observed in OS tumor tissue only. Furthermore, preclinical data demonstrated that this pathway activation is involved in OS progression. Therefore, the upregulation of EphA2 and its ligand in OS contributes to oncogenic signaling and might stimulate the OS metastasis process [62]. This target is also strongly expressed at the proteomic level as shown by PosthumaDeBoer et al. which observed that EphA2 was one of the most abundant and highly expressed proteins in OS cell lines and human OS samples. Furthermore, they underscored the significance of EphA2 expression in patients by establishing correlations with clinical parameters and demonstrating its association with poor overall survival [95]. Our group confirmed the pro-tumorigenic role of EphA2 in OS cells demonstrating that EphA2 silencing significantly reduced cell proliferation and migration [7]. We showed for the first time that the inhibition of EphA2 occurred after treatments with two receptor tyrosine kinase inhibitors, pazopanib and trametinib [7]. Using bioinformatic analysis, we explored the EphA2 gene expression level in 10 OS cell lines of the whole Cancer Cell Line Encyclopedia (CCLE) and its relation to patient characteristics and clinical outcomes in 88 OS samples deposited in Gene Expression Omnibus (GEO). We observed a higher expression of EphA2 in tumors with a higher Huvos grade than in the lower [13]. These findings allow us to speculate that, despite the good outcome of chemotherapy (high Huvos grade), persister cells surviving after/tolerant to chemotherapy and typically responsible for disease relapse, express higher levels of EphA2, suggesting that EphA2 could be a good target for second-line therapy, or its targeting could be combined with first-line chemotherapy to overcome drug resistance. In addition, significant upregulation of EphA2 in males compared to females was observed [13]. Considering that a worse prognosis was registered in male OS patients [96], this could be attributed to higher EphA2 expression. Overall, these data rationally support the exploration of the EphA2 targeting in OS.

### EphA2 in ewing sarcoma

ES is the second most frequent malignant bone tumor among children and adolescents with a median age at diagnosis of 15 years, slightly more common in males, and an incidence of 0.3 cases per 100,000 people per year, burdened by dismal prognosis in advanced stages [97]. The survival rate is 66% at 5 years and 20% at 5 years for poor responders [88]. Genetically, they are characterized by chromosomal translocation t(11;22)(q24;q29) in which the gene encoding for the RNA-binding protein EWS (EWSR1) is fused with the ETS transcription factor FLI1 resulting in EWSR1–FLI1 fusion gene. However, although rarely, few recurrent somatic mutations were found in ES, involving Cohesin Complex Component (STAG2), TP53, and Cyclin-Dependent Kinase inhibitor 2 A (CDKN2A) or else Kinase Insert Domain Receptor (KDR), Serine/Threonine Kinase 11 (STK11), DNA mismatch repair protein Mlh1 (MLH1), Kirsten rat sarcoma virus (KRAS), and Tyrosine-Protein Phosphatase Non-Receptor Type 11 (PTPN11) [98–100]. ESs are aggressive tumors, frequently displaying micrometastases at presentation [101]. These tumors exhibit a high sensitivity to chemotherapy and nearly 2/3 of the patients can be cured through multimodal treatment strategies, based on surgery and poli-chemotherapy with vincristine, doxorubicin, cyclophosphamide, and ifosfamide. Nevertheless, advanced/metastatic forms displayed poor outcomes [102]. ES family tumors do not benefit from established targeted therapies, emphasizing the imperative to uncover alternative therapeutic strategies. Recently, a phase I/II trial, conducted in a total of 85 patients not preselected for the ES molecular subtype, has shown good tolerability and limited activity of TK216, a small molecule inhibitor of EWS: FLI1 fusion protein inhibiting its function by preventing binding to RNA Helicase A [103].

Sáinz-Jaspeado et al. demonstrated the high expression of EphA2 protein in ES cell lines and patient samples investigating its association with the key membrane trafficking controlling protein CAV-1. CAV-1 contributes to angiogenesis in different tumors [104, 105]. In ES angiogenesis, CAV-1 increases EphA2 activation and signaling influencing its membrane localization [84, 106]. The formation of the EphA2/CAV1 complex also promoted the expression and secretion of bFGF, increasing tumor-induced migration of endothelial cells. These results suggest that in ES, EphA2-induced angiogenesis is dependent on CAV-1 [84]. In another study of the same group, EphA2 was reaffirmed as a significant contributor to the metastatic progression of ES owing to its involvement in cell signaling, mobility, and survival; moreover, the EphA2 phosphorylation at Ser897 was associated with ES aggressiveness [16]. Consistently, silencing EphA2 led to reductions in tumorigenicity, migration, invasiveness, and pulmonary metastatic progression in ES preclinical models. Finally, the knockdown of the metalloproteinase ADAM19, a downstream effector of EphA2 receptor signaling, negatively affects cell migration [16, 107].

Furthermore, our previous study investigated the mRNA expression levels of EphA2 in 12 ES cell lines from the CCLE and its correlation with tissue type, patient characteristics, and clinical outcomes within a cohort of 246 ES patients archived in GEO database. Specifically, we observed a significantly higher expression of EphA2 in tumor samples compared to normal tissues, with higher levels observed in male patients than females [13]. As for OS also for ES, male patients display a worse prognosis than females [108, 109]. These findings suggest that EphA2 might be related to more aggressive tumor behavior. Additionally, targeted inhibition of EphA2 resulted in a notable reduction of ES cell viability [13]. These experimental and computational findings collectively provide compelling evidence to support the hypothesis that targeting EphA2 overexpression may represent a viable therapeutic strategy for evaluation in advanced ES patients.

### EphA2 in chondrosarcoma

CS represents a malignant mesenchymal tumor and is the third most common among bone sarcomas across all age groups, yet it predominates in adults. Unlike OS and ES, it mainly occurs during adulthood in patients over the age of 40, with an average incidence of 0.2 cases per 100,000 people per year, both male and female [2, 3]. Most CSs arise as primary, low-grade, locally aggressive, non-metastasizing tumors (grade I) rather than high-grade (grades II-III). The histologic subtypes of CS include conventional, clear cell, mesenchymal, and dedifferentiated CS [3]. Patients with dedifferentiated CS are more likely to develop metastases and have a dismal prognosis with a 5-year overall survival of 7–24%. It is characterized by varied differentiated cells producing chondroid matrices, reflecting its high heterogeneity and association with intricate cytogenetic alterations [88]. Notably, mutations in Isocitrate Dehydrogenase (IDH) genes, specifically IDH1 and IDH2, are frequently observed, along with mutations in genes associated with cancer progression, such as TP53 [110]. CSs generally exhibit resistance to conventional chemotherapy regimens. Standard chemotherapy protocols yield poor results, and in cases in which surgical intervention becomes unviable, the prognosis is extremely poor. Recently, inhibition of IDH1 has shown some degree of activity [99]. Nevertheless, the clinical challenges posed by advanced CSs remain truly unmet in this context. The cornerstone of treatment for CS remains surgery and the result depends on the grade and location. Apart from low-grade CS of the extremities which are treated with extensive intralesional resection associated with local adjuvant treatment (high-speed burr, phenolization or cryotherapy, lavage with a high-pressure pulsatile system, and packing the defect with cement or bone graft). Generally, no adjuvant treatment is recommended in conventional CS due to its low sensitivity to radio and chemotherapy. Some retrospective reports suggest that mesenchymal CS is more chemo-sensitive, and may be considered for adjuvant or neoadjuvant therapy, mainly with Ewing-like regimens [111].

Zhang et al. evaluated the phosphorylation status of 42 RTKs in five CS cell lines showing that EphA2 was highly phosphorylated and constitutively activated in two of them [112]. Nevertheless, the EphA2 expression in CS tumor samples has not yet been extensively studied. In our previous work, we explored the EphA2 gene expression level in 4 CS cell lines belonging to the CCLE and its relationship with clinical and molecular features in a total of 102 CS patients from publicly available datasets. We observed a significantly higher expression of EphA2 in dedifferentiated CS samples with a worse prognosis than in better ones, and EphA2-specific inhibition impinged CS cell viability [13].

Considering these findings, there is a compelling rationale to pursue targeted therapy directed at EphA2 in advanced CS patients. Current therapeutic options for advanced CS remain notably inadequate, emphasizing the need for novel treatment strategies and EphA2 may represent a promising tool.

## Therapeutic strategies for targeting EphA2 in solid tumors

**Fig. 4:**
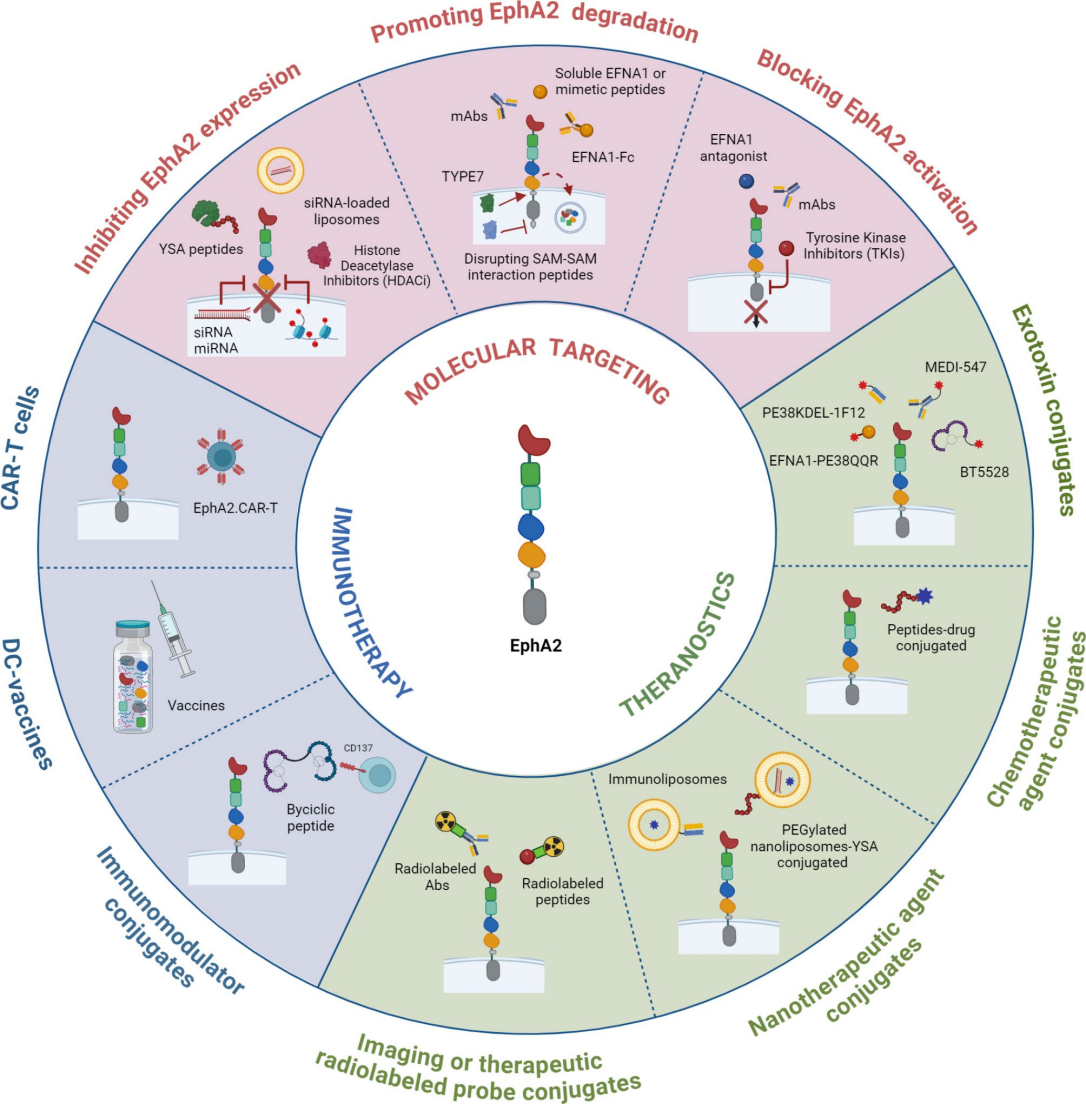
Schematic diagram summarizing the therapeutic targeting of EphA2 in solid tumors. EphA2 could be exploited as a molecular target ( pink ring): the disruption of its signaling through the inhibition of its expression by siRNAs, miRNAs, YSA-peptides, siRNA-loaded liposomes, and HDAC inhibitors; the promotion of its degradation by monoclonal antibodies, soluble EFNA1, mimetic peptides, EFNA1-Fc, disrupting SAM-SAM interaction peptides; or blocking its activation by EFNA1 antagonists, monoclonal antibodies, tyrosine kinase inhibitors; as a theranostic target (green ring) by using antibodies or peptides conjugates to exotoxins (MEDI547, BT5528, PE38KDEL-1F12, EFNA1-PE38QQR), chemotherapeutic agents (peptides-drug conjugated), nanotherapeutic agents (immunoliposomes or PEGylated nanoliposomes), or radiolabeled probes (radiolabeled antibodies or peptides); or as immunotherapeutic target (blue ring) by DC-vaccines, adoptive cell therapy approaches (CAR-T), or peptides conjugated with immunomodulators (bicyclic peptides). *(Created with BioRender.com)*

A plethora of molecular, genetic, epigenetic, biochemical, pharmacological, and immunotherapeutic strategies targeting EphA2 and its signaling pathways have been developed. This review explored a wide range of interventions, including small molecule inhibitors, monoclonal antibodies, drug or toxin-conjugated antibodies or peptides, chimeric antigen receptor T lymphocytes (CAR-T), and dendritic cell (DC) vaccines investigated in cancer preclinical models and clinical trials, providing valuable insights into their potential efficacy and translation applications also for bone tumors (Fig. 4).

### EphA2 as a molecular target

Among the different strategies that have been evaluated to disrupt EphA2 signaling, direct molecular targeting is by far the most investigated one. Indeed, several agents were shown to be able to bind the receptor and interfere with its tumor-promoting effects by different mechanisms such as inhibiting EphA2 expression, promoting its degradation, or blocking its activation [17](Fig. 4).

#### Inhibiting EphA2 expression

**Fig. 5:**
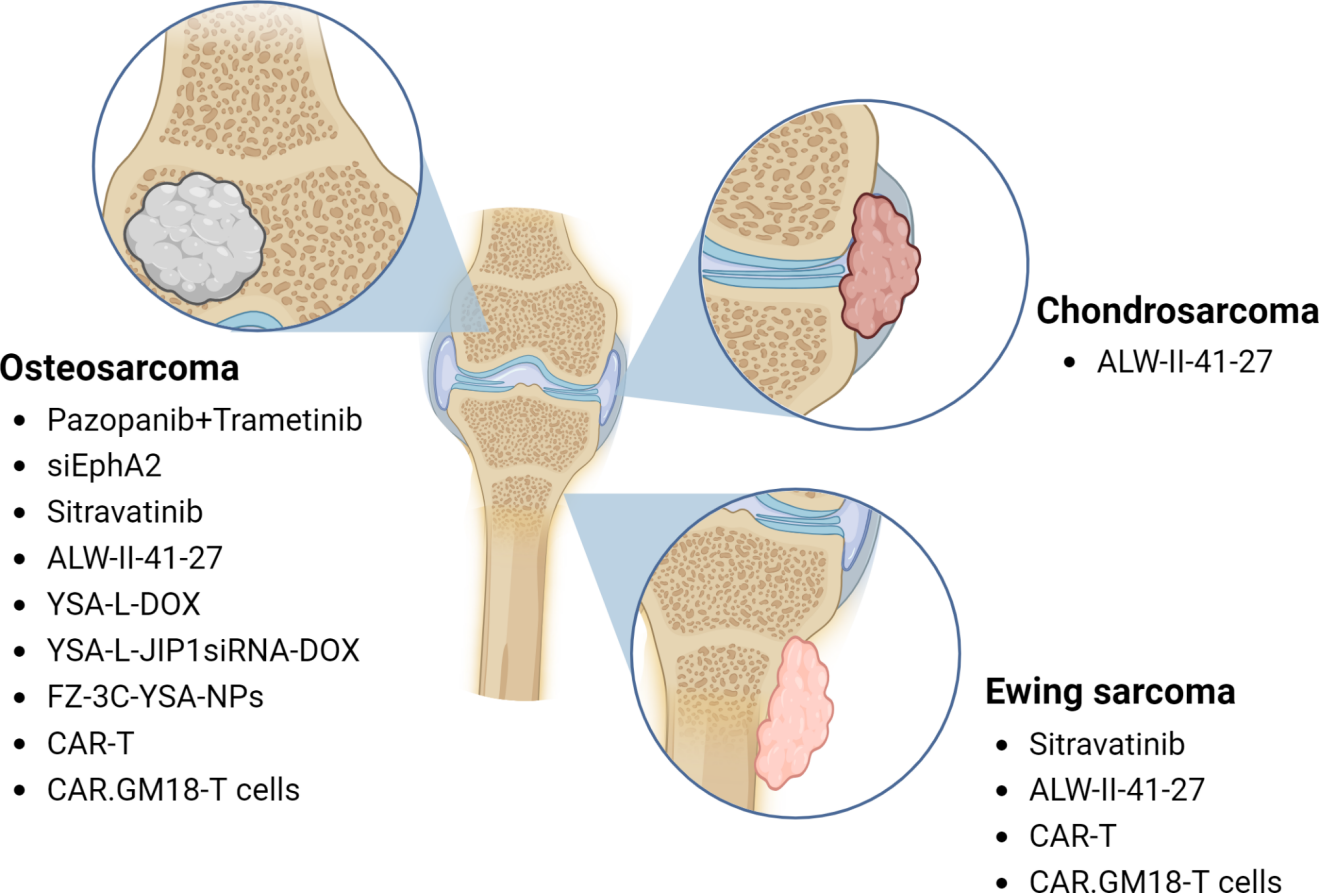
List of the therapies targeting EphA2 tested preclinically in osteosarcoma, chondrosarcomas, and Ewing sarcomas. *(Created with BioRender.com)*

Short interfering RNAs (siRNAs) for gene knockdown have been used for EphA2 silencing and suppressing its expression in human cancer cells. Several studies showed that decreasing the levels of EphA2, through silencing by specific siRNAs, significantly induces antitumor activity, reducing tumor growth and promoting apoptosis, both in in vitro [7] and in vivo models including lung cancer, breast cancer, and glioma [113–116]. We showed that the silencing of EphA2 reduced OS cell proliferation and migration [7] (Fig. 5). Moreover, Zhou et al. demonstrated that combining an EphA2 siRNA with either cisplatin, etoposide, or minustine hydrochloride significantly enhanced the antitumor effect against glioma cells [116]. However, although siRNAs have shown good results in vitro, their activity in vivo was limited by the difficulties of obtaining efficient delivery, cellular uptake, and good stability in body fluids [117]. Aiming to overcome this limitation and improve siRNA delivery, EphA2-specific siRNA was incorporated into neutral 1,2-dioleoyl-sn-glycerol-3-phosphatidylcholine (DOPC) liposomes resulting in an efficient tumor growth reduction in ovarian cancer mouse xenograft models when administered both as a single agent or in combination with paclitaxel [118]. Later, siRNA-DOPC was tested in murine and primate models, showing good safety and feasibility [119], and prompting a phase 1 clinical trial (NCT01591356). A different strategy to improve the EphA2 transcription silencing was proposed by Choi et al. who synthesized the p19-YSA fusion protein, composed of p19 RNA-binding protein and the EFNA1 mimetic YSA peptide as a siRNA delivery strategy. Specifically, YSA is a short amino acid sequence (YSAYPDSVPMMS) that binds with high affinity and selectivity to EphA2, while p19 can bind with high stability to siRNAs, protecting them from external RNAses [120, 121]. More recently, Oner et al. successfully developed a novel, safe, and efficient delivery system based on cationic solid lipid nanoparticles (cSLN) to enhance the bioavailability of EphA2-siRNAs in tumors. They used the dimethyldioctadecylammonium bromide (DDAB) to induce the reduction of the nanoparticle (NP) size and showed that siEphA2-loaded DDAB-cSLN displayed improved cellular uptake and EphA2 silencing [122].

Other than siRNA, miRNAs seem to be a good strategy to inhibit the EphA2 expression in many tumors. Different miRNAs are involved in the regulation of the EphA2 oncogene, varying according to the tumor type. At first, Tsouko et al. identified miR-200a as a direct repressor of EphA2 in triple-negative breast cancer, while Li et al. showed the antitumor effect of miR-26b on hepatocellular carcinoma cells [123, 124]. Subsequent research revealed that the overexpression of miR-200c downregulates EphA2 in malignant glioma, breast cancer, and lung carcinoma cells displaying efficient tumor-suppressive properties [125]. Similar tumor suppressive effects were observed with miR-141 in glioma cells, miRNA520d-3p and miR-302b in gastric cancers, and miR-519a on non-small cell lung cancer (NSCLC) cells [126–129]. Conversely, some miRNAs, such as miR-451a and miR-125a‐5p, naturally upregulate the expression of EphA2, making their inhibition a potential strategy to downregulate the oncogene expression. The reduction of miR-451a and miR125a-5p inhibits cell growth and metastasis in bladder carcinoma, and gastric cancer preclinical models, respectively [130, 131].

The overexpression of EphA2 stems from transcriptional activation through regulatory regions controlled by epigenetic regulators. EphA2 is associated with super-enhancers (SEs) activation in tumors [132, 133]. SEs are large clusters of enhancers that recruit multiple transcription factors and are implicated in the transcription of oncogenes [134]. Bioinformatic screening of Cancer Databases identified cancer-associated SEs by analyzing genomic regions with enriched histone 3 lysine 27 acetylation (H3K27Ac) marks. CRISPR technology targeting SE-associated EphA2 genes inhibits cell growth and metastasis. TCF7L2 and FOSL2 transcription factors were found to be recruited at SE-associated EphA2 loci during breast cancer progression [132]. Transcription factors exhibit cell-type-specific functions across different cancer types. Testicular nuclear Receptor 4 regulates EphA2 transcription in hepatocellular carcinoma and controls metastasis [135]. Changes in histone 3 lysine27 acetylation levels correspond to gene transcription regulation, with histone deacetylases (HDACs) playing a pivotal role in this process. HDACs are enzymes that modulate gene expression, making treatment with HDAC inhibitors a viable strategy to impede cancer progression. Notably, EphA2 expression was downregulated in advanced breast cancer following HDAC inhibitor treatment [115]. Furthermore, combining EphA2 and HDAC inhibitors enhanced the anti-tumor effects, underscoring the importance of identifying specific pathways in individual tumors to select optimal therapeutic approaches and develop personalized medicine [136].

#### Promoting EphA2 degradation

Soluble ligands or monoclonal antibodies (mAbs) that bind to EphA2 may disrupt oncogenic signaling by promoting receptor internalization and degradation [14]. Two independent studies showed that soluble EphA2 ligands in comparison with Fc-mAbs conjugated with EFNA1 induced EphA2 Tyr phosphorylation and proteasomal degradation, displaying tumor-suppressing properties in several tumor types including glioblastoma multiforme (GBM), gastric and breast adenocarcinoma [72, 137]. Additionally, Xu et al. demonstrated that mEA1-EYFP-H10, a fusion protein consisting of monomeric EFNA1 (mEA1) conjugate with an enhanced yellow fluorescent protein and linked to a supported lipid bilayer via a 10-histidine (H10) anchor, induced the Tyr phosphorylation of EphA2, and subsequent degradation [138]. Similarly, two short EFNA1 mimetic peptides, YSA and SWL, have been utilized to specifically target EphA2 LBD, inducing Tyr phosphorylation and signaling activation, albeit with less potency compared to soluble ligands and Fc conjugates [139]. Subsequent structural modifications of these peptides have led to improved derivatives capable of acting as both EphA2 agonist and antagonist and serving as pharmacological carriers [140].

Otherwise, Alves et al., take advantage of the acidic extracellular medium of solid tumors and created a highly soluble conditional peptide, called TYPE7, designed based on the sequence of the TM domain of EphA2, which binds the EphA2 endogenous domain acting as a molecular clamp that envelopes dimers of EphA2. TYPE7 reduced Akt phosphorylation and inhibited cell migration, mimicking the EFNA1 effect [141, 142]. Furthermore, the EphA2 SAM domain at the C-terminus facilitates interactions with regulators of receptor stability such as the lipid phosphatases Ship2 and the adaptor Odin. Ship2 decreased EphA2 endocytosis and consequent degradation, producing pro-oncogenic outcomes. In this setting, Mercurio et al. first discovered that the Ship2-SAM domain interaction promotes pro-oncogenic effects, identifying the peptide region involved in this interaction [143]. By computational and experimental approaches, they developed and tested several cyclic peptides that interfered with this complex and showed high serum stability and enhanced efficacy [144, 145]. Another strategy to modulate EphA2 activation regards the development of mAbs and other agonists acting as EFNA1 mimetics and eventually inducing receptor internalization and degradation [14]. For example, Carles-Kinch et al. identified EA1.2 mAb which recognizes specific epitopes of the EphA2 extracellular domain, inhibiting metastatic features on breast cancer cells, while sparing normal breast epithelial cells [146]. Different mAbs displayed similar results in several solid tumors. Coffman et al. showed that EA2 and B233 EphA2-specific mAbs promote EphA2 phosphorylation and degradation in breast and lung cancer cells, reducing cell growth in mice models, while Ansuini et al. developed and tested IgG25, another specific EphA2 mAb that promotes receptor endocytosis and subsequent degradation in pancreatic cancer in vitro and in vivo models, particularly reducing EphA2 protein levels and the phosphorylation of FAK on Tyr576 [147, 148]. Similarly, Jackson et al. generated an EphA2-specific fully humanized IgG1 mAb 1C1 able to induce receptor phosphorylation, internalization, and degradation [149]. DS-8895a is another new afucosylated humanized specific mAb which recognizes the extracellular juxtamembrane region of EphA2, interacting with both the full-length and truncated forms of the receptor. DS-8895a induces antibody-dependent cellular cytotoxicity, inhibiting tumor growth in both in vitro and in vivo models of breast and gastric cancers. Moreover, it potentiates the antitumor effect of cisplatin when administered in combination [150]. More recently, Sakamoto et al. produced three mAbs recognizing EphA2, showing that SHM16 inhibited migration and invasion of melanoma cells efficiently [151].

#### Blocking EphA2 activation

Agents binding to EphA2 or its ligand EFNA1 act as antagonists and suppress signaling, inhibiting Eph-Ephrin interaction or directly the receptor activity [17]. Dobrzanski et al. showed that the administration of EphA2/Fc soluble receptors, composed of EphA2 fused with the Fc region of human IgG1 antibody, functions as a decoy for activating ligands preventing their binding, and blocking the EphA2 signaling, thereby reducing tumor cell growth and invasiveness in preclinical models of pancreatic carcinoma [152]. Meanwhile, Ansuini et al. developed and tested a specific EphA2 mAb called IgG28 that impedes the binding to EFNA1, thereby impairing tumor vascularization in preclinical models of pancreatic cancer [148].

Another strategy was based on lithocholic acid (LCA), a secondary bile acid produced from chenodeoxycholic acid by colon bacterial activity. LCA is a competitive and reversible EphA2 antagonist that inhibits EphA2-EFNA1 interaction, blocking the receptor phosphorylation and activation; however, it does not discriminate between different combinations of Eph-Ephrin binding [153]. Several attempts were made to obtain and even increase the selectivity for EphA2 [154–157]. First, Giorgio et al. showed that LCA inhibited EphA2 phosphorylation in prostate and colon adenocarcinoma cell lines, without affecting other tested receptor tyrosine kinases [153]. They also discovered that the carboxylate group of LCA is critical for disrupting the EFNA1 ligand binding to EphA2 and identified the cholanic acid as a more competitive inhibitor than LCA [154]. Later, they synthesized a set of LCA derivatives that efficiently antagonized EphA2 in prostate cancer cells at low µM concentrations [155–157]. Finally, they designed and tested UniPR129, the L-homo-tryptophan conjugate of LCA that disrupted EphA2-EFNA1 interaction, inhibiting EphA2 activation and angiogenesis in prostate cancer cells [158]. Another derivative of 3β-hydroxy-D5-cholenic acid and L-tryptophan called UniPR1331 was active at low molecular concentration, showed good tolerability in GBM preclinical models, and potentiated the antitumor effect of bevacizumab when administered in combination [159]. Tognolini’s group efficiently demonstrated that the structural requirements for a small molecule to bind different receptors, among which Epha2, Farnesoid X receptor, and the G-protein-coupled receptor 5, are similar [160]. They selected and tested different Farnesoid X receptor agonists, among which Cilofexor, showing that they bind specifically and reversibly to EphA2 and interfere with EphA2 oncogenic phosphorylation in prostate adenocarcinoma cells [160].

Other researchers focused on tyrosine kinase inhibitors (TKI) and their potential to bind the EphA2 inner part, preventing its downstream signaling. Dasatinib, an oral multi-TKI, was the first studied in this context [161–163]. Chang et al. observed that, after EFNA1 stimulation, dasatinib inhibited EphA2 phosphorylation [163]. In addition, Buettner et al. showed that dasatinib inhibited the EphA2 tyrosine kinases activity, blocking migration and invasion, but not proliferation and survival, in human melanoma cell lines [161], while Ishigaki et al. observed that dasatinib exclusively inhibited the proliferation of EphA2-positive small-cell lung cancer (SCLC) cells, suggesting feasibility for clinical settings [114]. Dasatinib, either alone or in combination with chemotherapy, has been assessed in several clinical trials for advanced solid tumors (NCT00162214, NCT00792545), including SCLC (NCT00470054), squamous cell carcinoma (NCT00563290), endometrial cancer (NCT01440998) and in combination with radiotherapy in GBM (NCT00895960). Notably, this inhibitor has shown a broad range of targets making it challenging to interpret biological and clinical data [164–166]. However, these findings guided a chemical proteomics approach to designing and synthesizing new EphA2 inhibitors based on dasatinib structure. This approach seeks to exploit the ATP and the ribose pockets as binding epitopes in EphA2 kinase, improving its targeting profile. Compounds with an improved selectivity profile and potent anti-proliferative effect against GBM were obtained [167]. Similarly, Ho et al., using an orthogonal biological phenotypic screening approach, identified a group of newly synthesized benzylidene-indolinones able to inhibit multiple tyrosine kinases such as IGF-1R, Tyro3, and EphA2 phosphorylation. Among different candidates, they selected the most effective TKI which displayed a good safety profile and potent anti-proliferative, anti-migratory, and pro-apoptotic activities in hepatocellular carcinoma preclinical models [168]. Sitravatinib (MGCD516) is another multi-TKI inhibiting EphA2 [169]. Patwardhan et al. showed that this molecule promotes the blockade of RTK phosphorylation and induced tumor growth suppression in different sarcoma models, among which OS and ES, acting also in TKI-resistant models (Fig. 5) [170]. Based on these preclinical findings, a phase 1 clinical trial (NCT02219711) was conducted by Bauer et al. in patients with advanced solid tumors to evaluate its safety, pharmacokinetic, metabolism, pharmacodynamic, and clinical activity profiles, showing feasibility with only limited manageable side effects in these patients [171]. Several other clinical trials are currently ongoing to test the efficacy of sitravatinib in different types of cancers such as NSCLC (NCT03906071), liposarcoma (NCT02978859), squamous cell carcinoma (NCT03575598) and urothelial carcinoma (NCT03606174), as monotherapy or combination therapy.

Speaking of selective TKI targeting EphA2, ALW-II-41-27 showed promising antitumor effects in preclinical models of lung cancers, inducing time and dose-dependent apoptosis and tumor regression in NSCLC models, among which the EGFR-inhibitor-resistant ones, and inhibiting exclusively the proliferation of EphA2-positive SCLC models [113, 114, 172]. Furthermore, ALW-II-41-27 has been administered in combination with cetuximab reverting both primary and acquired drug resistance and resulting in proliferation inhibition, apoptosis induction, and tumor growth blockade in preclinical models of colorectal cancer [114]. This inhibitor is also effective in nasopharyngeal carcinoma cells, and in cervical cancer cells, reducing tumor growth, cell proliferation, migration, and invasion [173–175]. We also explored the effect of ALW-II-41-27 in OS, CS, and ES preclinical models, showing that it inhibited cell growth in a dose-dependent manner (Fig. 5) [13].

#### EphA2 as a target for theranostic applications

The overexpression of EphA2 in tumor cells and its relatively low expression level in normal tissue makes EphA2 an ideal target for tumor-specific delivery of chemotherapy or toxins while sparing healthy cells [14, 38]. Various types of antibodies-drug or peptides-drug conjugates have been developed and tested to hit EphA2-expressing tumor cells (Fig. 4).

#### Peptide/antibody exotoxin-conjugates

Derivatives of the highly cytotoxic exotoxin A of Pseudomonas aeruginosa were developed and conjugated with EFNA1 or with fragments of mAb recognizing EphA2 showing antitumor effects in preclinical models with limited side effects [176, 177]. In addition, Sakamoto et al. used the SHM16 mAb to deliver the saporin toxin in melanoma cells [151].

Another approach involves the conjugation of anti-EphA2 1C1 mAb with the toxin monomethyl auristatin phenylalanine (MMAF) via a stable maleimidocaproyl linker, known as MEDI-547 [149]. Upon EphA2 binding, this conjugate undergoes internalization and enzymatic cleavage, releasing MMAF that binds to tubulin, inhibiting its polymerization and inducing cell cycle arrest and apoptosis. MEDI-547 triggers activation of caspase-3/7 and cell death, EphA2 degradation, and tumor growth inhibition with minimal side effects in tumor preclinical models [178, 179]. Despite these encouraging results, in a phase I clinical trial (NCT00796055) testing MEDI-547, severe dose-limiting toxicity was observed arresting any further development of this strategy [180]. Then, novel EphA2-selective bicycle toxin-conjugates (BTC) were developed by Bicycle Therapeutics to avoid side effects. Mudd et al. used a phage display selection to generate a BTC with another potent microtubule inhibitor, the cytotoxin mertansine (DM1) via a cleavable linker, able to bind the EphA2-LBD at a low nanomolar range. After chemical optimization, they showed that it displayed potent antitumor activity and was well tolerated in xenograft models [181]. The most promising is BT5528 composed of the antimitotic agent MMAE linked via a chemical scaffold, capable of binding to EphA2 with high affinity and stability, avoiding off-target effect. Compared to MEDI-547, Bicycle toxins are much smaller and display a “fast-in and fast-out” mechanism of action exposing much less drug to the normal tissues [182]. MEDI-547 and BT5528 both carrying MMAE were compared: the BTC displayed potent antitumor activity avoiding hematologic adverse effects in animal models first, and in humans later, showing manageable side effects [182]. Moreover, the administration of BT5528, alone or in combination with nivolumab, in patients with solid tumors is ongoing in a phase I/II clinical trial (NCT04180371).

#### Peptide/antibody chemotherapy conjugates

The conjugation of peptides or antibodies that selectively bind EphA2 and induce its internalization provides a vehicle for targeting chemotherapeutic agents specifically to cancer cells sparing healthy tissues. Recently, Wang et al. conjugated the EphA2 agonist short peptide YSA with the cytotoxic agent paclitaxel (PTX) showing that it is significantly more effective than chemotherapy alone in a prostate cancer xenograft model [183]. Later, they optimized the drug-like properties of this delivery system by introducing non-natural amino acids, synthesizing, and testing two new PTX-conjugates EphA2 targeting peptides. In particular, dYNH-PTX displayed higher stability in mouse serum and significantly reduced tumor volume in prostate and renal cancer preclinical models [184]. Next, they investigated the chemical determinants responsible for the stability and degradation of these agents in plasma and introduced modifications to obtain more long-lived and more effective agents [185]. Despite their efficacy, these short peptides are often degraded and eliminated too rapidly in vivo. Therefore, Wu et al. developed 123B9-PTX, a novel tumor-homing agent that targets the EphA2-LBD, conjugated with PTX via a stable linker which displayed good efficacy in a pancreatic cancer xenograft and melanoma lung colonization and metastases models [186]. To overcome the limit due to the high concentrations required, they also developed a dimeric version of this compound (123B92–L2–PTX) which acted at nanomolar concentrations, targeting circulating tumor cells and inhibiting lung metastasis in breast cancer models [187]. The same strategy was adopted to conjugate gemcitabine with EphA2 targeting agents, developing YNH-L2-Gem and 123B9-L2-Gem, which showed good results in pancreatic cancer models [188]. Finally, more recently, Baggio et al. developed Targefrin-PTX, a novel agent targeting the EphA2-LBD that efficiently delivered to pancreatic cancers xenograft models reducing tumor volume and circulating tumor cells [189].

#### Peptide/antibody nanotherapeutic agent conjugates

The abovementioned peptides and antibodies can also be conjugated with PEGylated nanoliposomes and loaded with different drugs, such as the MEK inhibitor trametinib and the chemotherapeutic agent doxorubicin [190, 191]. This delivery approach allows drug release after internalization in tumor cells expressing EphA2, reducing circulating free-drug, and minimizing the off-target toxicity [190]. Fu et al. showed that the YSA-trametinib-loaded PEGylated nanoliposomes (YTPL) complex displayed the desired cytotoxicity against melanoma cells with higher uptake in vemurafenib-sensitive cells compared to its resistant counterpart [190]. Similarly, Haghilralsadat et al. showed that the YSA-doxorubicin-loaded PEGylated nanoliposomes (YSA-L-DOX) efficiently targeted the EphA2 receptor on human SAOS-2 OS cell line, promoting dose reduction and a higher cytotoxic activity (Fig. 5) [191]. Later, they developed a dual-targeted approach which consisted of targeting OS cell lines both extracellularly, against EphA2, and intracellularly against JNK-interacting protein 1 (JIP1) using YSA-PEGylated cationic liposomes loaded with doxorubicin and siRNA against JIP1 protein (YSA-L-JIP1siRNA-DOX). This strategy efficiently induced a reduction of JIP1 expression and the induction of apoptosis at the cellular level [192]. More recently, in collaboration with Carofiglio et al., we proposed to conjugate and with YSA peptide the iron-droplet zinc oxide (ZnO) nanoparticles coated with a 3 C lipidic shell and conjugated (FZ-3 C-YSA-NPs), to potentiate the specific targeting of OS. This construct triggers a specific cytotoxic effect that suppresses OS cell growth by increasing reactive oxygen species only when remotely activated by a mechanical pressure stimulation through ultrasound irradiation (Fig. 5) [193].

Another type of antibody-directed nanotherapeutics is MM-310. This is constituted by immunoliposomes loaded with the precursor form of docetaxel and conjugate with the anti-EphA2 scFv-3 peptide. Kamoun et al. tested the preclinical efficacy of MM-310 in bladder cancer xenograft models, both as a monotherapy and in combination with gemcitabine, showing that the combination improved tumor growth control [194]. Moreover, they studied the combination activity of MM-310 with the immune checkpoint inhibitors anti-PD1 and anti-PD-L1 in breast, colon, lung carcinoma, and in fibrosarcoma syngeneic mouse models, demonstrating the synergistic and immunomodulatory effects of the combination, mainly in breast cancer tumor models [195]. Based on these findings, a phase I clinical trial is ongoing to study the maximum tolerated dose and the safety profile of MM-310 in patients with advanced solid tumors, including soft tissue sarcoma (NCT03076372) [196].

#### Peptide/antibody imaging or therapeutic radiolabeled probe conjugates

Thanks to its high expression in both cancer cells and tumor vasculature compared to normal tissue, EphA2 can also be used as a target to deliver imaging agents for diagnostic and therapeutic purposes [197]. In this context, several specific probes consisting of radiolabeled peptides or antibodies binding EphA2 were developed for molecular imaging [198].

The first quantitative radioimmuno-positron emission tomography (PET) imaging of EphA2 was performed by Cai et al. in tumor-bearing mice, using the 64Cu-DOTA-1C1 compound, which consists of the 1C1 EphA2 antibody labeled with copper-64 (64Cu) through the chelating agent DOTA (1,4,7,10-tetraazacyclododecane N, N′,N″,N″′-tetra-acetic acid). This tracer showed promising results for clinical purpose investigation [199]. Similarly, Puttick et al. conjugated the 4B3 EphA2 antibody, labeled with 64Cu, with the chelating agent NOTA (1,4,7-triazacyclononane-1,4,7-triacetic acid) to perform PET imaging. They observed that 64Cu-NOTA-4B3 effectively delineates tumor burden, displaying both qualitatively and quantitatively better high-contrast images compared to other clinical standards imaging tools, in GBM mice models [200]. Later, Pyo et al. conjugated the same compound with another EphA2 antibody, E1, and observed that 64Cu-NOTA-E1 tracer displayed high tumor uptake and retention in human cancer prostate cells, rapid clearance in xenograft mice models and low background values in other tissues [201]. In addition, Burvenich et al. labeled another EphA2-specific antibody, DS-8895 A, with three different radio-isotypes: iodine-125 (125I), indium-111 (111In) and zirconium-89 (89Zr). They showed that 111In and 89Zr radio-conjugates displayed the highest uptake in EphA2-expressing tumors because of their entrapment inside the cell; on the contrary, 125I resulted in the lowest tumor uptake due to its internalization, translocation to lysosomes and subsequent degradation which release 125I-catabolites from the cells. Moreover, they observed that molecular imaging of DS-8895a tumor uptake, in particular using the [89]Zr-Df-Bz-NCS-DS-8895a compound, allows noninvasive measurement of EphA2 expression and determination of its saturation in xenograft models [202]. These considerations lead this compound in phase-I bio-imaging clinical trials, evaluating its dose assessment and patient response, showing that it is safe and well tolerated in patients with advanced solid tumors (NCT02004717; NCT02252211) [203, 204]. In the phase Ib study of DS-8895 A in patients with advanced/metastatic EphA2-positive cancers, no dose-limiting toxicities or treatment-related adverse events were reported. Observed non-drug related, treatment-emergent adverse events (grade ≤ 2) included fatigue (*n* = 3, 43%), diarrhea (*n* = 2, 29%), thrombocytopenia (*n* = 2, 29%), and decreased appetite (*n* = 2, 29%) [204].

Furthermore, Furukawa et al. recently developed and tested a novel imaging tracer to perform single-photon emission computed tomography (SPECT) of the EphA2 receptor. They conjugated the EphA2-230-1 mAb with the bifunctional chelator p-SCN-BnDTPA and labeled it with [111In], evaluating its affinity and pharmacokinetics. This [111In]In-BnDTPA-EphA2-230-1 tracer exhibited high tumor accumulation and successfully showed the tumor during the SPECT imaging on GBM mice models [205].

In analogy, radiolabeled peptides could be used for diagnostic imaging and eventually targeted radiotherapy. For instance, Pretze et al. synthesized [18 F]AFP-SWL, a new radiolabeled peptide consisting of the EphA2 specific SWL peptide conjugated with the bioorthogonal radiolabeling building block [18 F]AFP (1-(3-azidopropyl)-4-(3-fluoropropyl)piperazine). They were the first to perform a radio-pharmacological characterization of this radiotracer, evaluating its metabolic stability [206]. However, a more promising tracer was developed by Liu et al. conjugating technetium-99 m (99mTc) with the same peptide, resulting in the 99mTc-HYNIC-SWL tracer which displayed rapid blood clearance by renal excretion in lung cancer and melanoma in vivo preclinical models [207]. Moreover, Furukawa et al. developed [123I]ETB, a SPECT imaging tracer for the EphA2 receptor which consists of an ALW-II-4127 EphA2 inhibitor derivative (ETB), labeled with gamma-emitting iodine-123 (I-123), showing that it selectively bound and inhibited EphA2 and also revealed an efficient tumor uptake in GBM mice models [208].

### EphA2 as a target for immunotherapy

The extraordinary developments in immunotherapy make the preferential expression of EphA2 in solid tumors an attractive possible target for anticancer immunotherapy. Indeed, thanks to its membrane expression, EphA2 can be a good target for vaccines and adoptive cell therapy approaches, such as the CAR-T strategy [209] (Fig. 4).

#### EphA2 redirected CAR-T

Nowadays, a very promising immunotherapy strategy is based on autologous T lymphocytes extracted from patient blood samples, genetically engineered ex vivo to express artificial chimeric antigen receptors (CAR) recognizing a specific tumor antigen and then re-infused into the patient to obtain a T-cell dependent tumor cell killing. The definition of a selective and tumor-specific target is crucial to avoid side effects [210]. CAR-T cells specifically directed against EphA2 have been developed and studied against different tumors, such as NSCLC, esophageal squamous cell carcinoma (ESCC), GBM, and pediatric bone sarcomas [211–214]. At first, Chow et al. developed EphA2-specific T cells constituted by EphA2-CAR with a CD28-ζ endodomain which induced a potent anti-cancer activity on GBM cells and the regression of GBM xenograft in SCID mice, prolonging their survival [213]. Then, Yi et al. generated and compared different EphA2-specific CAR-T against GBM models, showing that T cells expressing both the endo-domains CD28.ζ and 41BB.ζ CARs with short spacers efficiently improve the CAR-T cell function, compared to incorporating the 41BB domain into CD28.ζ CARs [215]. Similarly, a second generation of efficient specific EphA2.CAR-T with the co-stimulatory receptor 4-1BB was developed and tested in NSCLC and ESCC in vitro and in vivo models [211, 212]. Hsu et al. tested EphA2 CAR-T therapy against OS and ES, demonstrating its anti-tumor activity in metastatic murine models both in vitro and in vivo **(**Fig. 5**)** [214]. Moreover, Lange et al. recently developed GM18, a novel CAR consisting of the GM-CSF receptor extracellular domains and the IL18 transmembrane domains, that links CAR-T cell activation to MYD88 signaling. It was developed to sustain the CAR-T cell function after repeated exposure to tumor cells, by activating an antigen-dependent autocrine loop. They showed that CAR.GM18-T cells significantly increase the expansion and production of cytokines in vitro and induce tumor regression in ES and OS in vivo models, compared to standard CAR-T (Fig. 5) [216]. Finally, a recent advancement in CAR-T therapy involves the development of TanCAR-T cells to enhance the treatment of GBM both in vitro and in vivo. TanCAR-T cells are engineered with a tandem arrangement of IL13 (4MS) and EphA2 scFv, as well as both targets when present on cancer cells. Importantly, TanCAR-T cells are programmed to spare normal cells that express only the IL13Ra1/IL4Ra receptor, thereby minimizing off-target effects and enhancing the specificity of the therapy [217].

In the first clinical trial with EphA2-redirected CAR-T cells, the reported toxicities in the first 3 treated patients were pulmonary edema with cytokine release syndrome in 2 out of 3 patients. This might be due to the physiological expression of EphA2 by lung epithelial cells [218]. Unfortunately, our relatively little knowledge on EphA2 expression by adult healthy tissues further complicates our ability to predict which other organ sites could be affected by on-tumor, off-target side effects.

#### DC-vaccines

Despite its physiological expression in normal tissues, the relatively low distribution of the EphA2 receptor in adult cells, compared to cancer cells, renders it an almost ideal target for vaccine-based strategies. In this scenario, DC-based vaccines emerge as a promising therapeutic approach in cancer immunotherapy since they promote antitumor response taking advantage of each patient’s immune system [219]. Indeed, DCs are proficient antigen-presenting cells specialized to capture, process, and present antigens on major histocompatibility complex molecules [220]. Moreover, DCs play key roles in the communication between innate and adaptive immunity and are responsible for the antigen-specific adaptive immune response initiation stimulating both helper T cells and cytotoxic T lymphocytes (CTLs) that recognize specifically tumor cells [221]. The first evidence to support the potential of EphA2-based vaccine therapies was realized by Hatano et al., they demonstrated that EphA2-derived peptide vaccination promoted immunity and induced therapeutic anti-tumor effects in mice models. Furthermore, they observed that this vaccination effectively prevented tumor establishment or growth in EphA2-positive syngeneic glioma, sarcoma, and melanoma models and that EphA2 vaccines could also be directed to EphA2-negative target cells, targeting tumor-associated vascular endothelial cells expressing EphA2, as different tumor-associated antigens [222, 223]. In addition, to improve the CTL-mediated immune response against glioma cells, Chen et al. developed and tested a novel vaccine containing EphA2883–891 peptide and LIGHT plasmid in HLA-A2 transgenic mice. They observed that this vaccine induced a robust cellular anti-tumor immunity against U251 glioma cells and inhibited tumor growth [224]. As well, Yeung et al. demonstrated that an increased expression of tumor-associated antigens such as EphA2, IL-13Rα2, and Survivin, allowed the basis for the utilization of an established multiple peptide vaccine in pediatric and adult ependymomas [225]. Therefore, Pollack et al. worked on a pilot study of subcutaneous vaccinations using these glioma-associated antigens epitope peptides emulsified in Montanide-ISA-51. They showed good tolerability and immunogenic activity with preliminary evidence of efficacy ascribing this as a promising strategy [226]. The results obtained with vaccines in glioma tumors were later confirmed in other models. Yamaguchi et al. evaluated the immunotherapy efficacy of DCs pulsed with EphA2-derived peptide on murine MC38 colon cancer models. They demonstrated that this strategy inhibited tumor growth in EphA2-positive colon cancer xenografts but not in EphA2-negative melanoma ones. Moreover, they observed that natural killer cells, but not CD4 + and CD8 + T lymphocytes, were necessary for immunizations and the vaccine had long-term anti-tumor immunity [227, 228]. Additionally, the researchers observed heightened tumor-specific CTL activity in both colon cancer and melanoma mice models [229]. Therefore, EphA2-DCs and EphA2-NPs vaccines warrant further studies in selected EphA2-expressing tumors. Furthermore, in a recent phase II trial, Storkus et al. demonstrated that dasatinib is a potent adjuvant in specific vaccination against overexpressed and non-mutated tumor blood vessel antigens, including EphA2. The active recruitment of T cells in tumor sites lowered myeloid-derived suppressor cells and regulatory T cell abundance extending the patient’s overall survival. This vaccination combined with dasatinib was safe and resulted in immunologic and clinical responses in melanoma patients [230].

#### Peptide/antibody immunomodulator conjugates

Another strategy to take advantage of EphA2 specificity is the stimulation of the immune cells exclusively at the tumor site, exploiting a specific binding to EphA2 tumor cells and immune cells infiltrating the nearby microenvironment. BCY12491, a tumor-targeted immune cell agonist (TICA) exemplifies a new class of fully synthetic immunomodulators consisting of two bicyclic peptides, constricted each other, targeting respectively EphA2 as tumor antigen, and a co-stimulatory molecule CD137 or 4-1BB, a member of the tumor necrosis factor receptor superfamily. Upadhyaya et al. showed that BCY12491 is a highly specific and potent immune cell stimulator through CD137 agonism, in EphA2-overexpressing tumors that avoid systemic activation. Preclinical data confirmed that BCY12491 showed potent EphA2-dependent immunomodulatory activity in vitro and induced local tumor regression, complete responses, immunogenic memory, and significant modulation of the tumor immune microenvironment in preclinical syngeneic mouse models [231]. These findings provide a strong rationale to further develop the first-in-class Bicycle TICAs to potentially treat EphA2-expressing cancers.

## Conclusions

In conclusion, targeting EphA2 in bone sarcomas is a promising therapeutic strategy due to its highly tumor-specific expression and pivotal role in tumor progression and metastasis. The development of advanced drug delivery systems, such as NP-based carriers, antibody-drug conjugates, small molecule inhibitors, and gene therapy approaches, enhances the precision and efficacy of EphA2-targeted therapies. These methods not only improve drug delivery to the tumor site but could also help minimize systemic toxicity. However, given the lack of data on EphA2 protein expression in adult normal tissues [38], considerations on potential on-target, off-tumor effects should be considered cautiously. Despite its benefits, targeting EphA2 and its delivery system might lead to off-target effects, immune reactions, and toxicity. Disrupting normal cellular functions of EphA2 and the potential development of resistance are additional concerns. Hematologic toxicity, affecting blood cell counts and bone marrow function, is also a risk. Ongoing research and clinical trials are crucial to optimize these therapies, addressing the challenges and ensuring safe and effective treatment options for patients with bone sarcomas. EphA2 overexpression in bone tumors compared to normal tissues and its involvement in key tumor processes (e.g., proliferation, migration, and angiogenesis) supports its validity as a therapeutic target. EphA2 targeting can work synergistically with other treatments, potentially lowering the risk of resistance development, which typically ensues with all single-agent targeted treatments. Continued investigation into the mechanisms of EphA2 in cancer biology and the refinement of delivery methods will be key to realizing the full therapeutic potential of EphA2 targeting bone sarcomas. Dedicated clinical trials of EphA2 targeting in the diverse histotypes of each bone sarcoma are still missing, and the study of EphA2 protein expression in the different bone sarcoma subtypes can be useful for predicting the efficacy of EphA2 targeting strategies. These strong preclinical and early clinical studies of multifaceted targeting of EphA2 give rise to encouraging results of efficient novel therapeutic strategies against EphA2-expressing tumors. The distinct expression patterns of EphA2 and its pivotal role in promoting tumor progression render this protein an attractive target for therapy across various cancers. This includes advanced bone sarcomas which remain challenging to treat and lacks effective therapeutic options so far. These findings open the way toward novel therapeutic avenues for addressing these aggressive, rare, and currently “drug-orphan” diseases.

## Data Availability

No datasets were generated or analysed during the current study.

## Abbreviations

EphA2: Ephrin Type-A Receptor 2
OS: Osteosarcoma
ES: Ewing sarcoma
CS: Chondrosarcoma
RTKs: Receptor tyrosine kinases
Eph: Erythropoietin-producing hepatocellular
LBD: Ligand-binding domain
EGF: Epidermal growth factor
FN: Fibronectin
TM: Transmembrane
TK: Tyrosine kinase
SAM: Sterile alpha motif
RBD: Receptor-binding domain
GPI: Glycosylphosphatidylinositol
Tyr: Tyrosine
EFNA1: Ephrin-A1
TNF: Tumor necrosis factor
ERKs: Extracellular signal -regulated kinases
FAK: Focal adhesion kinase
Rho G: Ras homolog gene family, member G
CAF: Cancer associated fibroblast
CAV-1: Caveolin-1
LMW-PTP: Low molecular weight phospho-tyrosine phosphatase
bFGF: Basic FGF
CCLE: Cancer cell line encyclopedia
GEO: Gene expression omnibus
EWSR1: RNA-binding protein EWS
STAG2: STAG2 cohesin complex component
TP53: Tumor protein 53
CDKN2A: Cyclin-dependent kinase inhibitor 2 A
KDR: Kinase insert domain receptor
STK11: Serine/threonine kinase 11
MLH1: DNA mismatch repair protein Mlh1
KRAS: Kirsten rat sarcoma virus
PTPN11: Tyrosine-protein phosphatase non-receptor type 11
IDH: Isocitrate dehydrogenase
CAR-T: Chimeric antigen receptor T lymphocytes
DC: Dendritic cell
DOPC: 1,2-Dioleoyl-sn-glycerol-3-phosphatidylcholine
cSLN: Cationic solid lipid nanoparticles
NP: Nanoparticle
NSCLC: Non-small cell lung cancer
DDAB: Dimethyldioctadecylammonium bromide
SEs: Super-enhancers
HDACs: Histone deacetylases
GBM: Glioblastoma
mAbs: Monoclonal antibodies
mEA1: Monomeric EFNA1
LCA: Lithocholicacid
TKI: Tyrosine kinase inhibitors
SCLC: Small-cell lung cancer
MMAF: Monomethyl auristatin phenylalanine
BTC: Bicycle toxin-conjugated
PTX: Paclitaxel
YTPL: YSA-trametinib-loaded PEGylated nanoliposomes
YSA-L-DOX: YSA-doxorubicine-loaded PEGylated nanoliposomes
PET: Positron emission tomography
DOTA: 1,4,7,10-Tetraazacyclododecane N, N′,N″,N″′-tetra-acetic acid
NOTA: 1,4,7-triazacyclononane-1,4,7-triacetic acid
125I: Iodine-125
111In: Indium-111
89Zr: Zirconium-89
SPECT: Single-photon emission computed tomography
AFP: 1-(3-azidopropyl)-4-(3-fluoropropyl)piperazine
99mTc: Technetium-99 m
ETB: Epha2 inhibitor derivative
I-123: Iodine-123
TICAs: Tumor-targeted immune cell agonists
ESCC: Esophageal squamous cellcarcinoma
CTLs: Cytotoxic T lymphocytes
